# Joint Damage and Neuropathic Pain in Rats Treated With Lysophosphatidic Acid

**DOI:** 10.3389/fimmu.2022.811402

**Published:** 2022-02-04

**Authors:** Jason J. McDougall, Allison R. Reid

**Affiliations:** Departments of Pharmacology and Anaesthesia, Pain Management and Perioperative Medicine, Dalhousie University, Halifax, NS, Canada

**Keywords:** neuropathic pain, arthritis, sex differences, histopathology, animal model

## Abstract

Joint pain is a complex phenomenon that involves multiple endogenous mediators and pathophysiological events. In addition to nociceptive and inflammatory pain, some patients report neuropathic-like pain symptoms. Examination of arthritic joints from humans and preclinical animal models have revealed axonal damage which is likely the source of the neuropathic pain. The mediators responsible for joint peripheral neuropathy are obscure, but lysophosphatidic acid (LPA) has emerged as a leading candidate target. In the present study, male and female Wistar rats received an intra-articular injection of LPA into the right knee and allowed to recover for 28 days. Joint pain was measured by von Frey hair algesiometry, while joint pathology was determined by scoring of histological sections. Both male and female rats showed comparable degenerative changes to the LPA-treated knee including chondrocyte death, focal bone erosion, and synovitis. Mechanical withdrawal thresholds decreased by 20-30% indicative of secondary allodynia in the affected limb; however, there was no significant difference in pain sensitivity between the sexes. Treatment of LPA animals with the neuropathic pain drug amitriptyline reduced joint pain for over 2 hours with no sex differences being observed. In summary, intra-articular injection of LPA causes joint degeneration and neuropathic pain thereby mimicking some of the characteristics of neuropathic osteoarthritis.

## Introduction

Peripheral nerve damage is a prominent feature of joint diseases such as osteoarthritis (OA) and rheumatoid arthritis (RA). For example, Lanzillo et al. found that around 60% of RA patients in their study had impaired nerve conduction velocities and 75% of biopsied nerves showed mild to moderate damage (1). Ahmed et al. reported that 54% of RA patients in their cohort reported severe pain, about a third of which was neuropathic in nature (2). The sequelae of joint neuropathy are multifarious including impairment of neurovascular control mechanisms, altered proprioception, and the emergence of neuropathic pain (3). In its most severe form, joint sensory denervation leads to Charcot arthropathy the characteristics of which include extreme bone loss, osteophyte formation, and synovitis. This structural damage partly arises due to a lack of neurosensory feedback from the joint culminating in altered gait and abnormal loading patterns during movement. Afferent nerve depletion has also been observed in the joints of patients with OA and this phenomenon is a consequence of transient articular inflammation destroying peripheral nerves (4, 5). A significant proportion of the remaining nerves innervating arthritic joints appear as tortuous neuromas which are a likely source of neuropathic pain. A similar pattern of joint neuropathy has been described in animal models of OA in which the arthritis was either chemically-induced (6, 7), surgically-induced (8), or naturally occurring (9).

Peripheral neuropathy can arise due to a focal injury of the nerve such as following a mechanical insult, or it may occur in response to an atypical neuroimmune response. Following joint injury, articular nerves take on a damaged appearance which resembles peripheral neuropathy (10). The mechanisms and molecules responsible for this joint nerve destruction are obscure, but lysophosphatidic acid (LPA) is a leading candidate. This endogenous bioactive lipid is the product of the hydrolysis of lysophosphatidylcholine by an enzyme called autotaxin (11). The production of synovial LPA is elevated in patients with OA (12) and RA (13) suggesting that extracellular LPA may be involved in neuropathic joint diseases. In addition to increased autotaxin activity in arthritis (14), LPA levels could also rise as a result of impaired catabolism by lipid phosphate phosphatases (15). Acutely, LPA causes the gradual decline in myelin basic protein production which is a key element in maintaining myelin integrity (16). More long-term, LPA interferes with the expression of genes necessary for neuronal survival, axon guidance, and cyto-architecture (17). The resulting demyelination leads to aberrant nerve morphology and firing patterns which manifests as neuropathic pain.

In joints, LPA is known to contribute to the pathogenesis of arthritis and the development of neuropathic pain. Autotaxin expression in fibroblast-like synoviocytes has been shown to be five times greater in RA patients (18) with the highest expression occurring in regions close to damaged cartilage (19). Transgenic arthritic animals which lack ATX or LPA_1_ receptors show reduced levels of joint damage, pannus formation, and synovitis suggesting that LPA is a major driver of inflammatory joint disease (20, 21). Similar anti-arthritic effects were observed in response to pharmacological blockade of LPA_1/3_ receptors with Ki16425 (22). It has previously been reported that intra-articular injection of LPA caused demyelination and peripheral sensitization of joint nociceptors which was also blocked by Ki16425 (12). The nociceptive effect of LPA involves increased gating of the nociceptor voltage-gated sodium channel Na_v_1.8 as the selective antagonist A-803467 reduced pain and afferent firing in the rat LPA model (23). This effect was more pronounced in female rats.

The aim of the present study was to characterise the LPA model further by comparing joint pain and pathology between the sexes. The sensitivity of the model to the classic neuropathic pain drug amitriptyline was also assessed.

## Methods

Male (250-300g) and female (200-225g) Wistar rats (Charles River, Quebec, Canada) were housed in pairs in ventilated racks with free access to water and standard chow pellets. Ambient temperature was set at 21 ± 1°C with a 12 hour light: 12 hour dark cycle. All animals were allowed to acclimate for 1 week prior to being used in experimental protocols. All interventions had received prior approval from the Dalhousie University Committee on the Use of Animals which adheres to the guidelines set out by the Canadian Council on Animal Care.

### The Lysophosphatidic Acid Model of Joint Neuropathy

Deep anaesthesia was induced by gaseous inhalation of isoflurane (2-5% in 100% O_2_ at 1L/min) which was confirmed by areflexia to noxious toe pinch and corneal blinking. The right stifle (knee) joint was shaved and swabbed with 100% ethanol. Lysophosphatidic acid (LPA; 100µg in 5% ethanol/saline) was injected as a 50µl bolus into the joint cavity and the hindlimb extended/flexed for 30s to disperse the fluid throughout the knee. Ipsilateral joint diameters were measured weekly using digital callipers (Mastercraft, Vonore, TN, USA) oriented in a medio-lateral plane across the joint midline.

### Joint Histopathology

At day 28 following LPA injection, knee joints were harvested and fixed in 4% paraformaldehyde for one week, then subsequently dehydrated in 70% alcohol, and decalcified using 5% formic acid (4-5 days). Knees were then halved in the frontal plane and embedded in paraffin. Tissue was sectioned in 160µm steps and three sections per joint were stained with toluidine blue. Joint damage was scored by a blinded observer using the OARSI guidelines and the average for three sections was recorded (24). Histopathology scores were based on inflammation (inflammatory cell infiltration and oedema), pannus formation (extent of synovial hypertrophy), cartilage damage (loss of toluidine blue staining as an indicator of proteoglycan loss), and bone resorption (percentage of bone loss in the joint). Each parameter was scored from 0 (normal) to 5 (severe) to give a total possible score of 15.

### Pain Behavioural Assessment

Animal pain behaviour was determined before (baseline) and then weekly following LPA injection. Secondary allodynia was assessed by von Frey hair algesiometry. Rats were placed in a clear Perspex compartment with a mesh wire floor which allowed access to the plantar aspect of the hindpaws. After an initial acclimation period (~10min), von Frey hair filaments of specific bending force were applied to the plantar surface of the hindpaws using the Dixon up-down method (25). Flexed filaments were held in place for 3s and any reaction to the tactile stimulus was noted (*i.e.* paw withdrawal, licking, or shaking of the foot). If no response occurred, the next stiffer filament was applied up to a cut-off of 15g. If a response was noted a lower force filament was applied. The 50% withdrawal threshold was determined using the following formula: 10^[^ *^Xf^* ^+^ *^k^* ^δ]^/10,000; where *Xf* = value (in log units) of the final von Frey hair used, *k* = tabular value for the pattern of the last 6 positive/negative responses, and δ = mean difference (in log units) between stimuli.

### Amitriptyline Treatment

Day 28 LPA animals were briefly anaesthetised with isoflurane and the neuropathic pain drug amitriptyline (7.5mg/kg dissolved in 0.9% saline vehicle) was injected intraperitoneally. This dose was based around previous pain assessment studies in rats (26). Pain behaviour was subsequently measured at 0, 30, 60, 120 and 180min following drug administration.

### Drugs and Reagents

1-Oleoyl-LPA sodium salt (Abcam) was dissolved in a 5% ethanol/saline solution to a final concentration of 100 µg/50 µL. Amitriptyline was procured from Sigma-Aldrich (St. Louis, MO, USA) and dissolved in 0.9% saline.

### Statistics

All data were expressed as mean ± SEM. Data were tested for Gaussian distribution by the Kolmogorov–Smirnov test. All time courses were analysed using a repeated measures 2-way analysis of variance (ANOVA) with a Holm-Sidak *post hoc* multiple comparisons test for individual timepoints. Joint histopathology scores were analysed using a 2-paired Mann-Whitney test. Spearman’s rank coefficient was used to determine any correlation between joint pathology and hindpaw withdrawal threshold following LPA treatment. A P value less than 0.05 was considered statistically significant.

## Results

**Figure 1:**
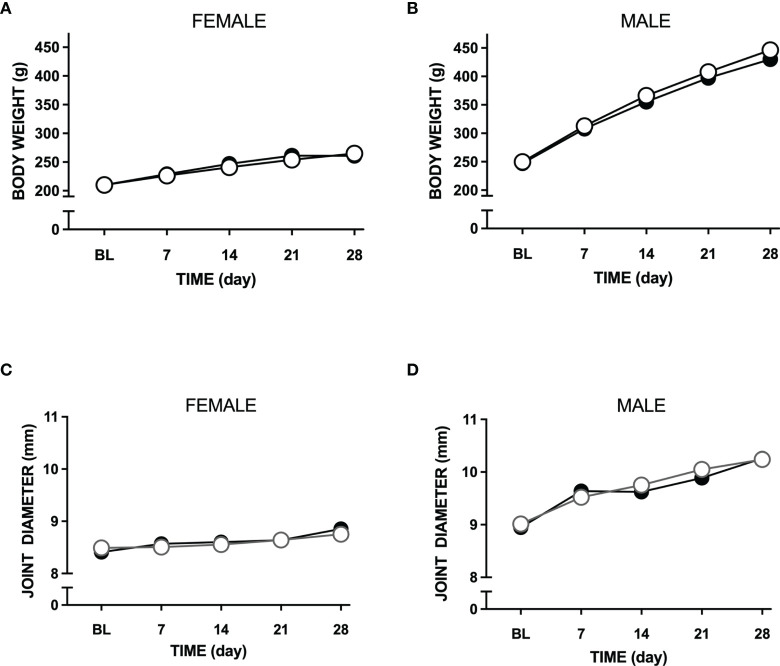
Effect of LPA on body weight and joint oedema. Intra-articular injection of LPA (100µg) had no effect on body weight in either female **(A)** or male **(B)** rats. Animal growth progressed normally indicating that there were no systemic adverse effects of LPA treatment. Knee joint diameter was also unaffected by LPA treatment over the 28 day time period **(C, D)** confirming a lack of inflammatory oedema in the model. Data are means ± S.E.M.

Over the 28-day testing period, LPA treatment had no significant effect on body weight in female (P=0.59) or male (P=0.26) animals (**Figures 1A, B**). In addition, ipsilateral joint diameter was also unaffected by LPA injection in both sexes (P=0.42 female; P=0.69 male. **Figures 1C, D**).

### Joint Histopathology

**Figure 2:**
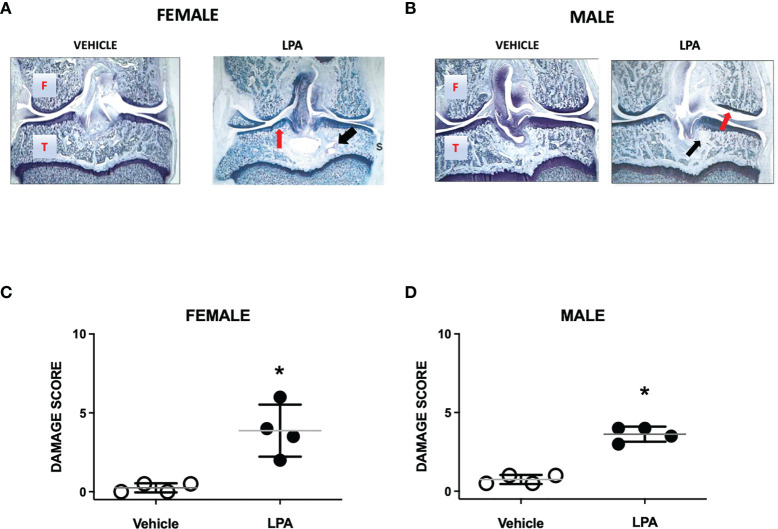
Joint histopathology in male and female LPA-treated knees. Representative photomicrographs of female **(A)** and male **(B)** knee joints 28 days following treatment with LPA. Compared to vehicle control, LPA caused proteoglycan loss (red arrows) and focal lesions to subchondral bone (black arrows). (Magnification: X16. T, Tibia; F, Femur; S, Synovium). Damage scores in female **(C)** and male **(D)** knees were significantly higher in LPA-treated joints compared to control. (*P < 0.05, unpaired Student t-test; *n*=4 animals/group). Data are means ± S.E.M.

At day 28, female and male joints showed mild damage compared to vehicle-treated animals. Pathological changes included diffuse synovitis, multifocal loss of proteoglycans, mild chondrocyte death, and focal bone resorption (**Figures 2A, B**). A bone cyst near the tibial insertion of the cruciate ligament was observed in one of the female samples. Compared to vehicle control, total knee joint damage score was significantly increased in both females and males (P<0.05; Mann-Whitney test; *n*=4 animals/group, **Figures 2C, D**). There was no statistical difference in LPA-induced disease severity between female and male knee joints (P=0.99).

### Effect of LPA on Joint Pain

**Figure 3:**
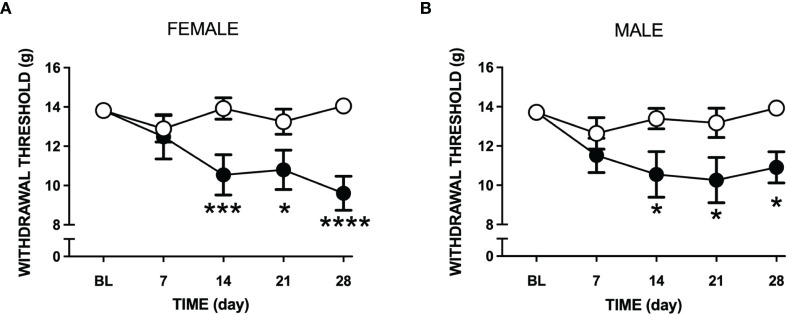
Changes in tactile sensitivity to von Frey hair filaments following LPA treatment. Mechanosensitivity in vehicle (open circles) and LPA-treated (closed circles) female **(A)** and male **(B)** rats over 28 days following intra-articular injection of LPA. Secondary allodynia gradually developed over the time course and was still present at day 28. (*P < 0.05; ***P < 0.001; ****P < 0.0001 two-way ANOVA with Holm-Sidak *post hoc* multiple comparisons test; *n*=12-24 animals/group). Data are means ± S.E.M.

Intra-articular injection of LPA caused a reduction in paw withdrawal threshold in both male and female rats (P<0.0001; 2-factor ANOVA; *n*=12-19 female, *n*=12-24 male). This secondary allodynic effect occurred on days 14-28 after LPA injection (**Figures 3A, B**). At day 28, female withdrawal thresholds fell by about 30% compared to baseline while male mechanosensitivity decreased by approximately 20%. There was no statistical difference in pain response between female and male animals over the 28 day time course (P=0.96).

**Figure 4:**
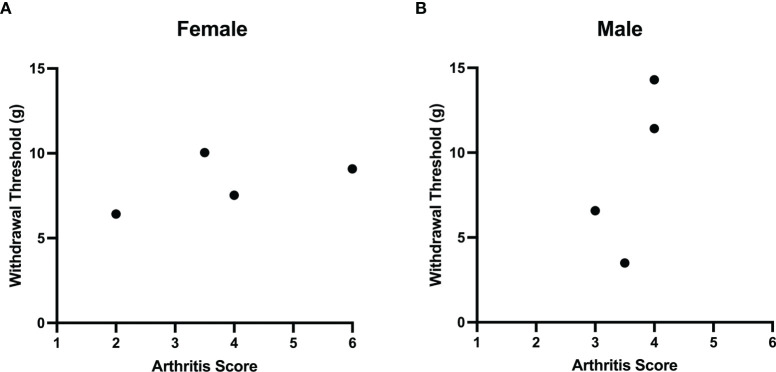
Lack of correlation between LPA-induced joint damage and mechanonociception. A comparison of arthritis score and secondary mechanical allodynia failed to demonstrate any correlation between these two parameters in both female **(A)** and male **(B)** rats treated with LPA. (Females: Spearman’s rank coefficient r = 0.4, P = 0.75, *n* = 4; male: Spearman’s rank coefficient r = 0.7, P = 0.33, *n* = 4).

Comparisons of joint histopathology scores and baseline von Frey hair withdrawal thresholds revealed no correlation between arthritis severity and pain sensitivity in female (r=0.4, P=0.75; Spearman’s rank coefficient) and male (r=0.7, P=0.33) LPA-treated animals (**Figure 4**).

### Effect of Amitriptyline on LPA-Induced Pain

**Figure 5:**
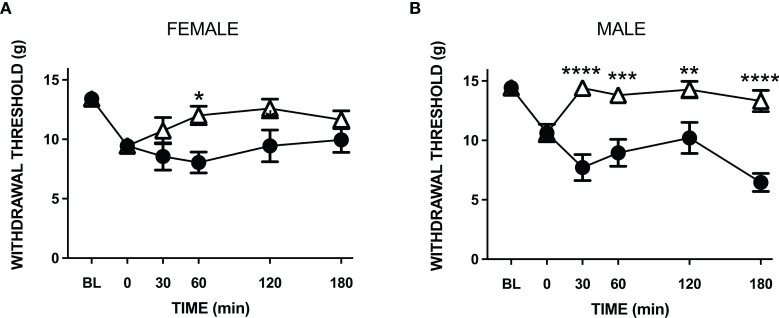
Anti-nociceptive effect of amitriptyline in LPA-injected neuropathic joints. Compared to vehicle control (closed circles) systemic administration of amitriptyline (open triangles) reduced secondary allodynia in both female **(A)** and male **(B)** LPA animals. (*P < 0.05; **P < 0.01; ***P < 0.001; ****P < 0.0001 two-way ANOVA with Holm-Sidak *post hoc* multiple comparisons test; *n* = 9-10 animals/group). Data are means ± S.E.M.

Treatment of LPA animals with amitriptyline significantly increased hindpaw withdrawal thresholds in both male and female rats (P<0.001, *n*=9-10 animals/group; **Figures 5A, B**). The analgesic effect of the drug lasted approximately 2 hours and there was no significant difference in amitriptyline effects between female and male animals (P=0.60).

## Discussion

Peripheral neuropathy is a contributing factor to the development of OA pain in approximately 30% of patients. This observation is reshaping how OA pain is managed as previous reliance on anti-inflammatory pain medications has proven to be inadequate in many patients. The higher incidence of neuropathic pain in females suggests that the neurophysiological underpinnings of pain development are sex-specific. The mediators and mechanisms responsible for joint neuropathy are still being worked out; however, LPA has emerged as a leading contender (12). The aim of the present study, therefore, was to compare the effect of LPA on joint pathology and pain in male and female rats.

Twenty-eight days after local injection of LPA into the knee, male and female joints developed a significant deterioration in structural integrity compared to control. Animal growth was unaffected by LPA treatment indicating a lack of any adverse systemic effects. Lesions in the subchondral bone and articular cartilage were observed while synovitis was minimal suggesting a degenerative type of arthritis. It has previously been shown that intra-articular injection of LPA causes demyelination and damage to joint afferents which can be blocked by the LPA_1/3_-receptor antagonist Ki16425 (12). This sensory neuropathy would likely impair articular vasoregulation and compromise protective proprioceptive feedback mechanisms which help stabilise the joint. A regulated blood supply is critical for maintaining joint homeostasis and promoting tissue healing. These vasomotor control mechanisms are lost following joint denervation indicting that an intact nerve supply is critical for joint health (27). Similarly, OA joints are associated with proprioceptive deficits resulting in increased joint laxity and abnormal loading patterns (28). It has been postulated that loss of neurological control in joints could lead to degenerative changes consistent with OA (3) and this phenomenon could be contributing to the pathological changes observed here.

Acutely, LPA may be promoting arthritis by affecting cellular processes in joint tissues directly. *In vitro* studies using fibroblast-like synoviocytes found that LPA stimulated the release of the pro-inflammatory cytokines interleukin-6 and interleukin-8 (29). These cytokines are known to evoke angiogenesis, neutrophil recruitment, and hyperaemia and can therefore be considered pro-arthritic. In other studies, the LPA_1_-receptor antagonist LA-01 was able to block the production of inflammatory mediators, intra-articular macrophage infiltration, and bone destruction in a mouse model of RA (21). These transient pro-inflammatory effects of LPA occurred acutely and therefore do not correspond to the pathological changes described in our chronic experiments. With the later time points tested in the present study, there was no joint oedema and synovitis was minimal. While the acute, pro-inflammatory effect of LPA could produce early local degenerative changes in the joint, the chronicity of the model and its associated pain is more likely due to neuropathy and the loss of joint neural regulation.

Damage to joint nerves invariably leads to the manifestation of neuropathic pain. Intra-articular injection of LPA produced a reduction in hindpaw withdrawal threshold and this effect was not sex-specific. Furthermore, there was no statistical correlation between the level of joint degeneration and pain sensitivity which is consistent with what has been previously reported in other models (30). The tactile secondary allodynia observed in LPA-treated rats was blocked by the neuropathic pain drug amitriptyline supporting the notion that the type of pain produced by LPA was due to neuronal damage. Demyelination of axons innervating the joint could lead to the exposure of additional Na_v_ channels and the emergence of ectopic nerve firing. It has previously been reported that LPA causes an increase in spontaneous and evoked firing of joint nociceptors and this was due to an increase in Na_v_1.8 gating activity (23). In contrast to the current study which found no sex differences, O’Brien et al. found that LPA-induced pain behaviour was greater in female rats; however, this was probably due to a lower dose of LPA being used than in the current study. Autotaxin is an enzyme that cleaves a choline group from lysophosphatidylcholine to form LPA and this enzyme is gaining interest as a target to treat neuropathic pain. Autotaxin levels in the synovial fluid of RA and OA patients correlates with disease severity (18, 21, 31) and pharmacological blockade of the enzyme in rodent models of OA resulted in significant analgesia (32). Thus, the autotaxin-LPA pathway appears to be an attractive approach to treating joint neuropathic pain.

In summary, intra-articular injection of LPA in rats culminated in degradation of the joint and neuropathic pain. There were no observable sex differences in pathology or pain which is probably due to the higher dose of LPA used in this study. Thus, intra-articular injection of LPA offers a useful model of joint neuropathy which is amenable to testing neuropathic pain drugs.

## Data Availability Statement

The raw data supporting the conclusions of this article will be made available by the authors, without undue reservation.

## Ethics Statement

The animal study was reviewed and approved by Dalhousie University Committee on the Use of Animals.

## Author Contributions

JM conceived the study, managed the experiments, helped analyse data, and wrote the manuscript. AR carried out the experiments, helped analyse the data, and helped write the manuscript. All authors contributed to the article and approved the submitted version.

## Conflict of Interest

The authors declare that the research was conducted in the absence of any commercial or financial relationships that could be construed as a potential conflict of interest.

## Publisher’s Note

All claims expressed in this article are solely those of the authors and do not necessarily represent those of their affiliated organizations, or those of the publisher, the editors and the reviewers. Any product that may be evaluated in this article, or claim that may be made by its manufacturer, is not guaranteed or endorsed by the publisher.
